# Coaxial nanofiber scaffold with super-active platelet lysate to accelerate the repair of bone defects†

**DOI:** 10.1039/d0ra06305c

**Published:** 2020-09-29

**Authors:** Zhipeng Huang, Wantao Wang, Qinglong Wang, Taylor Hojnacki, Yanli Wang, Yansheng Fu, Wenbo Wang

**Affiliations:** The First Affiliated Hospital of Harbin Medical University 23 You Zheng Street Harbin 150001 China wenbowang1967@163.com; Department of Cancer Biology, Abramson Family Cancer Research Institute, Perelman School of Medicine, University of Pennsylvania 421 Curie Blvd. Philadelphia PA 19014 USA; Tianqing Stem Cell Co., Ltd. Jubao Second Road, Science and Technology Innovation City, Songbei District Harbin 150000 China

## Abstract

To develop biocomposite materials with the local sustained-release function of biological factors to promote bone defect repair, coaxial electrospinning technology was performed to prepare a coaxial nanofiber scaffold with super-active platelet lysate (sPL), containing gelatin/PCL/PLLA. The nanofibers exhibited a uniform bead-free round morphology, observed by a scanning electron microscope (SEM), and the core/shell structure was confirmed by a transmission electron microscope (TEM). A mixture of polycaprolactone and sPL encapsulated by hydrophilic gelatin and hydrophobic l-polylactic acid can continuously release bioactive factors for up to 40 days. Encapsulation of sPL resulted in enhanced cell adhesion and proliferation, and sPL loading can increase the osteogenesis of osteoblasts. Besides, *in vivo* studies demonstrated that sPL-loaded biocomposites promoted the repair of skull defects in rats. Therefore, these results indicate that core–shell nanofibers loaded with sPL can add enormous potential to the clinical application of this scaffold in bone tissue engineering.

## Abstract 2

Coaxial electrospinning three-dimensional scaffold and its release various biological factors after filling the bone defect to induce adhesion and proliferation of osteoblasts on the nano scaffold.

## Graphical abstract

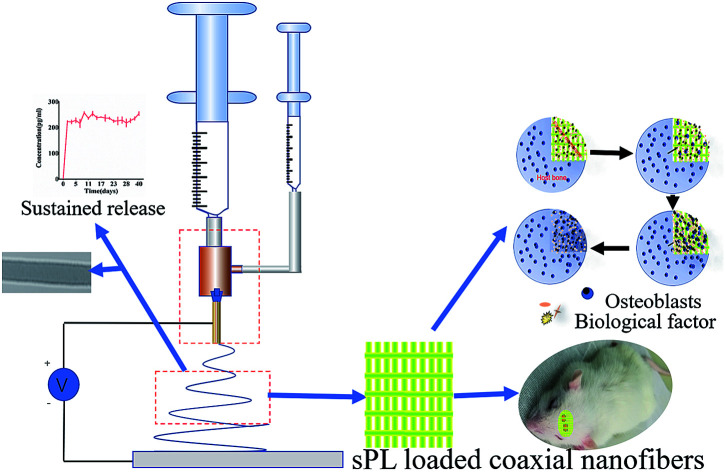

## 1. Introduction

The treatment of bone destruction disorders, such as large bone defects associated with comminuted fractures or bone tumor resection and nonunion caused by fractures, is a major challenge for orthopedic surgeons. Complete and stable reconstruction of bone is an ideal treatment strategy for bone destruction.^1^ Tissue engineering scaffolds are the basis for the development of artificial bone and the key to bone tissue regeneration.^2^ The combination of high porosity, flexibility and mechanical properties make this type of fiber the material of choice for various biomedical applications. Bioscaffolds are a promising drug delivery materials because they can provide supporting scaffolds for growing cells and tissues.^3^ In addition, the scaffolds fill the bone defects during bone regeneration.^4^

Platelet-rich plasma (PRP) is effective in accelerating the healing of various tissue damage (*e.g.*, skin trauma,^5,6^ bone/cartilage defects,^7,8^ and tendon/ligament injury^9,10^). Super-active platelet lysate (sPL) is a rapid release of high-concentration bioactive factors in platelets based on the patented platelet-efficient induction–activation culture technique. However, naturally prepared sPL cannot be stored for a long time as a liquid and must be repeatedly injected in the treatment of bone defects. In addition, the fluidity of liquid sPL may lead to heterotopic ossification, which cannot provide enough space for the restoration of bone defects.

Electrospinning technology provides a solution to this problem, it is a simple, versatile technique for producing micron and nanoscale polymer fibers, which is considered a superior drug delivery system.^11^ More importantly, the morphology of electrospun nanofiber scaffolds (NFS) is similar to ECM. Fibers with high surface area to volume ratio and high porosity can promote cell adhesion and proliferation and maintain cell phenotypic characteristics.^12^ Unfortunately, the high temperature generated during electrospinning can reduce the biological activity of growth factors.^8^ The polymer nanofibers produced by the coaxial electrospinning technology can avoid the contact between the core layer and the surface layer, and protect the growth factor in the core. Moreover, the release rate of coaxial nanofibers is lower than that of uniaxial nanofibers, which is conducive to long-term release.^13^ Therefore, the core–shell transfer system ensures the sustained release of core drugs or growth factors in the fiber. Here, we use the coaxial electrospinning technology to prepare the three-dimensional nanofiber scaffold. Poly(ε-caprolactone) (PCL) with sPL as the core of the nanofiber, and a combination of poly-l-lactic acid (PLLA) and gelatin as the shell. Gelatin, it provides special attachment, migration and proliferation in different tissue regeneration applications, and is often used as part of electrospinning composite materials.^14^ PLLA and PCL are widely used to promoted osteogenic gene expression by electrospinning due to biocompatibility and biodegradability.^15^

The osteogenic effect of the scaffold was evaluated *in vitro* and *in vivo* experiments. We aimed to (1) evaluate whether the nanofiber scaffold can be used to store and control the release of sPL-derived growth factors; (2) evaluate the optimal concentration of sPL nanofiber scaffolds for cell adhesion, proliferation, and osteogenesis; (3) evaluate the ability of the nanofiber scaffolds to promote healing of the rat skull defects, further guiding and stimulating the body's inherent healing response of cell and tissue defects. These evaluations can prove that the scaffold synthesized by coaxial electrospinning and the bio-friendly material carrying sPL has great clinical application potential and also provides an idea for the treatment of bone defects.

## 2. Results and discussion

### 2.1. Synthesis and characteristics of sustained-release bioactive factor nanofiber composite scaffolds

**Fig. 1:**
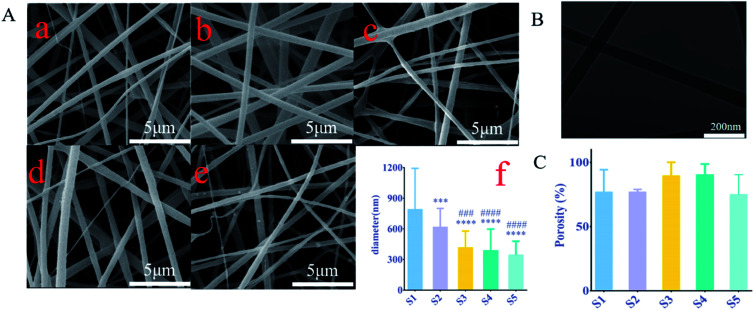
(A) SEM images of sPL materials with different concentrations. (a) S1, (b) S2, (c) S3, (d) S4, (e) S5, (f) fiber diameter of different materials; (B) coaxial structure of nanospun fibers revealed by TEM; (C) porosity of loaded sPL composite scaffolds; data are expressed as mean ± SD (*n* = 3).***P < 0.001, ****P < 0.0001, compare to S1;^###^*P* < 0.001, ^####^*P* < 0.0001, compare to S2.

Studies have shown that the electrostatic attraction between biopolymers and the conductivity of solvent systems are two factors driving the electrospinning process, any factor that increases the charge density will result in the formation of fibers with narrow diameters.^16^ The surface of all the electrospun nanofibers was smooth and the fiber diameter was uniform in each sample (Fig. 1A(a–e)). The fiber diameter distribution was between 92 nm and 1596 nm in 5 samples (Fig. 1A(f)) with an average of 515.6 nm. The fiber diameter of S1–S5 decreased with the increase of sPL. Compared with S1, the S2 fiber diameter of S3–S5 is significantly reduced (*P* < 0.001). However, there was no statistically significant change between S3–S5 (*P* > 0.05). It can be seen that sPL affected the diameter of nanofibers to a certain extent; the difference in diameter distribution of nanofiber supports may be due to the polarity of electrospun scaffold materials.

Changes in fiber diameter also led to changes in pore size, as can be seen from Fig. 1A, where the pore diameter of the nanofibers increased. As can be seen from Fig. 1A, the size of the pore size is related to the fiber diameter. This was a necessary condition for cell migration and adhesion. Studies show that^17^ bone conduction in bone grafts depended not only on the properties of the material but also on the porous structure, which was a morphological property.

Pore structure parameters included pore shape, porosity, pore size, and pore interconnectivity, where pore size and interconnection of pores were directly related to bone mineralization and angiogenesis.^18,19^ To verify the core–shell structure of the fiber (Fig. 1B), the coaxial structure of the nanofibers was detected by a transmission electron microscope scan.

According to the Archimedes principle, the porosity of S1, S2, S3, S4, and S5 was 76.9 ± 14.18%, 76.9 ± 1.4%, 89.5 ± 8.56%, 90.4 ± 6.7%, and 80 ± 7.62%, respectively (Fig. 1C). These values have closely resembled the porosity of cancellous bone.

The porosity of three-dimensional fiber changed as the sPL content increased; however, there was no significant difference in the porosity between all the composite brackets. This porosity was established during the path of the polymer jet from the tip to the collector where water droplets present in the atmosphere condense on the surface of the polymer solution resulting in a print on the surface. Porous structures can also be explained by the phase separation behavior of spinodal decomposition. Karageorgiou V. *et al.*^17^ compared the porosity and pore size of different biomaterials for the extent and type of bone formation *in vivo* and *in vitro* experiments, and they found that a decrease in porosity would enhance *in vitro* osteogenesis. However, for *in vivo* experiments, higher porosity promoted the recruitment and penetration of bone tissue cells around the scaffold as well as vascularization, which was beneficial for osteogenesis.

**Fig. 2:**
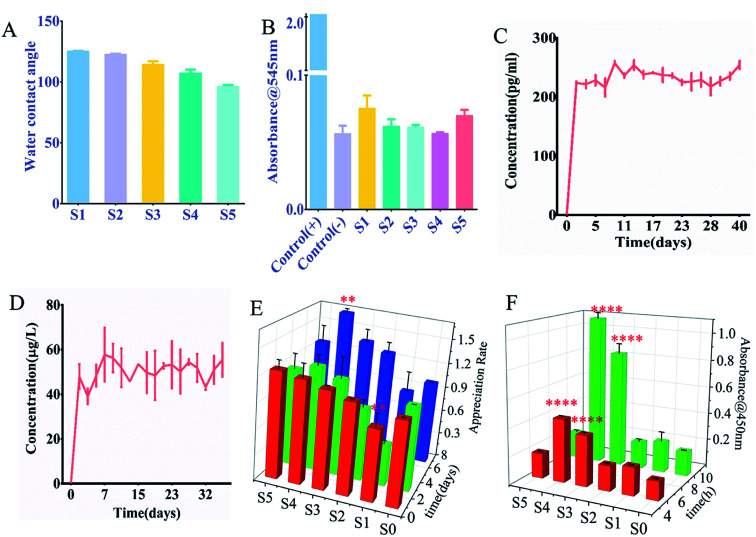
(A) Water contact angle of coaxial electrospun nanofibers; (B) absorbance of coaxial electrospun nanofibers after contact with blood; (C) cumulative release curve of VEGF growth factor; (D) cumulative release curve of IGF growth factor; (E) the proliferation activity of osteoblasts detected by CCK-8 method after 1, 3, and 7 days of different scaffold cultures. (F) Number of adherent osteoblasts after 4 and 8 hours of culture. All data were labeled as mean ± standard deviation, *n* = 6, **P < 0.01,***P < 0.001,****P < 0.0001, compared to S0.

The hydrophilicity of the nanofiber surface was another important factor in the application of tissue engineering scaffolds, as it affected the attachment and proliferation of cells. The nanofiber scaffold hydrophobicity was evaluated by measuring the contact angle. Fig. 2A and S1† showed the results obtained for a nanofiber scaffold with sPL. The surface water contact angle (hydraulic metric) of the electrospun PLLA stent was 128.2 ± 2.3°, which limited its application in tissue engineering.^20^ Therefore, we mixed gelatin and PLA for the outer surface of the coaxial spinning material. This study found that as the sPL content increased the value of contact angle decreased, indicating that the hydrophobicity of the material was reduced. Scaffolds S1–S5 were placed in a glass dish containing a sufficient amount of water. As shown in Fig. S2,† S1 exhibited a slower water absorption rate, which reached maximum water absorption after 60 min; all S2–S5 stents all exhibited rapid water absorption properties, and the maximum water absorption was achieved after being in the water for 15 minutes. The maximum water absorption rate increased slightly with the increase of sPL between scaffolds S1 and S3; however, between S3–S5, sPL increased but the water absorption rate decreased, and the scaffold grew over time. The maximum water absorption rate did not change drastically, demonstrating that the stent exhibited strong water absorption properties. This is consistent with the porosity of the nano-scaffold and blending technology, which is considered to be a cost-effective technique for controlling the material and physicochemical properties of polymer biomaterials.^21^ The nanofiber scaffolds prepared herein had excellent water absorption properties, which was different from the hydrophobic properties of the three-dimensional nanofiber scaffolds prepared by Ding,^22^ Greiner.^23^ This is different from the measurement of contact angle, which is approximate because the contact angle measures the result of 5′′ contact of water on the rotating film, and the hydrophilicity is the result of measuring the complete water absorption of the spinning material.

The three-dimensional fiber scaffold carrying sPL was placed in PBS for 1, 3, 5, 7, and 9 days, and the degradation of the fibers was measured, As shown in Fig. S3a,† the fiber almost unchanged in the first 5 days, but the fiber degraded significantly on the 7^th^ day. On the 14^th^ day, the nanofibers were scanned by SEM (Fig. S3B†), the surface of the fibers had degraded slightly, and some of the fibers had some condensation on the surface with decreased pore size. Documentary proof PLLA is a slowly crystallized, hydrophobic semi-crystalline polymer with the ability to degrade l-lactic acid, which is a substance that is non-toxic to humans. PLLA is biocompatible, easy to process, and produces non-toxic degradation products.^24^ PLLA does not produce a significant inflammatory response, and the rate of degradation *in vivo* is similar to the rate of degradation *in vitro*. The degradation time is very slow,^25^ so the stent has a longer degradation time.

The hemolysis test absorbance was measured at 545 nm for the scaffold material, as shown in Fig. 2B the OD value of the nanofiber was much lower than that of the salt saline group and there was no significant difference in the OD value for each group of nanofibers. Fig. S4† shows the hemolysis experiment image, and the same trend was exhibited. The deionized water is shown in red, and other materials and saline appearing clear. Also, all materials are non-toxic. Therefore, although the increase in sPL resulted in higher biological efficacy, there was no significant hemolytic activity. The hemolytic activity was quantified by the percentage of hemoglobin released by the lysed red blood cells, and the hemolysis rates of S1–S5 were 0.93%, 0.33%, 0.256%, 0.266%, and 0.0094%, respectively, 100% hemoglobin cleavage relative to deionized water. According to ISO 10993-4 standard, these hemolysis rates were much less than 5%, which was in line with clinical device application standards. This may be attributed to the fact that the materials we use are all non-toxic. First of all, the materials we use, gelatin, PCL, and PLLA are all biocompatible, biodegradable, and approved by the FDA, which have played an important role in bone reconstruction surgery.^26–28^ Secondly, the solvent we use is 2,2,2-trifluoroethanol, which has high volatility and has been volatilized during the production of nanofiber membranes. Finally, sPL is of human origin, and the production process is all pollution-free steps such as centrifugation.

### 2.2. Representative bioactive factors in sPL release from nanofiber composite scaffolds

To accurately assess the release kinetics of the growth factors within the sPL scaffolds, we evaluated the release of two major growth factors in sPL, VEGF and IGF; which were contributed to osteogenesis. Experiments were performed on the nanofibers of S4, and Fig. 2C and D show the release of VEGF and IGF, respectively. The cumulative release profile of growth factors indicates that the protein in sPL-NFS was maintained for 40 days. VEGF and IGF reached the highest after the onset of release on day 1 and reached the plateau in the next maintenance equilibrium. Although the two growth factors had differential fluctuations during release, the amount they released was very low, which might be due to factor release differences with repeated measurements. Statistical analysis found no statistically significant difference between releases.

Based upon literature reviews, there were two different ways of releasing a loaded drug: diffusion-based release (diffusion from a polymer matrix) and degradation-based release (diffusion on a barrier). In these two release systems, the concentration gradient inside the material was the driving force of diffusion. In the early stage, the explosive release occurred due to the high concentration gradient, but with the change of concentration gradient, the late release dropped sharply. The distance was the determining factor influencing the release curve.^29,30^ In a degradation-based delivery system, the local barrier separated the drug reserved in the polymer from the release environment, and the concentration of the drug in the local tissue was related to the overall amount of the drug. In such systems, a nearly constant release rate and linear release profile can be accomplished.^31^ Therefore, the balance among the diffusion of biologically active factors, the concentration of biomaterials and the concentration of bioactive substances was the key to the sustained release of biologically active factors. Coaxial electrospinning techniques can be used to create a type of release system that diffused through a barrier or was based on erosion. In this study, we used PCL as the core with a combination of gelatin and PLLA as the outer shell. Further results suggested that the growth factor release of the three-dimensional fiber scaffold carrying sPL was a sustained release.

### 2.3. sPL-loaded nanofiber composite scaffold promotes osteoblast adhesion and proliferation

Cell activity was observed by CCK-8 on the three-dimensional scaffolds (S1, S2, S3, S4, S5) at 4 h, 8 h, 1 day, 3 days, and 7 days. It can be seen from Fig. 2E that three-dimensional fibers with sPL can promote the proliferation of osteoblasts. The results showed that S4 significantly proliferated on day 7 compared to S0 (*P* < 0.01). Compared to S1 and S3, S4 experienced significant proliferative effects on the 1^st^ day of cell and scaffold action. Compared to day 1, S4 had significant proliferation (*P* < 0.05) on days 3 and 7. On day 7, cell number increased on S1, S2, S3, and S5 compared to the S0 fiber scaffold, but increased significantly on S4 (*P* < 0.01).

The cells were seeded on the fiber scaffold for 4 h and 8 h, and OD values were measured to evaluate whether there were cells present on the scaffold. As shown in Fig. 2F, the cells showed obvious adhesion on the scaffold containing sPL compared with the S1 (sPL-free scaffold). When sPL content reached 37.5%, cell adhesion was significantly enhanced (*P* < 0.001). The addition of sPL reduced the fiber diameter, increased the porosity and hydrophilicity, and changed the scaffold structure making it more conducive to cell adhesion. This was consistent with the results of the Hutmacher *et al.* study.^32^ Highly porous matrices promoted progenitor cell migration, proliferation, and differentiation to induce bone regeneration. At the same time, one advantage of using coaxial fibers in biomedical applications, namely as a stent, could combine appropriate mechanical properties with highly biocompatible materials. Zhang *et al.*^33^ demonstrated that pure PLLA electrospun materials had very good flexibility and high elongation at break. In Komur *et al.*'s materials,^34^ fibers had higher ultimate tensile strength characteristics as PCL concentration increased.

**Fig. 3:**
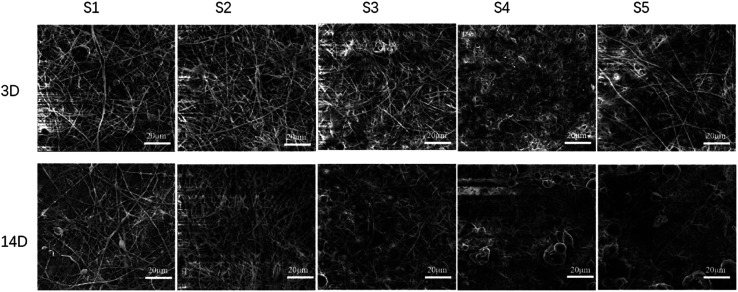
Cell co-culture with sPL three-dimensional fiber scaffold after 3 and 14 days by SEM.

SEM was used to observe the adhesion and growth of the cells on the scaffold. As shown in Fig. 3 and S5,† the cells adhered to the scaffold of S0–S5, indicating that the cells grew well. An increase of sPL increased the number of cells present on the scaffold with the highest number of cells on S4, and the filaments were observed on the 3 days indicating that fiber degradation was not obvious. On the 7^th^ day, as the sPL increased, cell adhesion increased as well, and the agglomeration of the fiber scaffold increased compared with S0. At 14 days, the cells were covered with a fiber surface observed at the larger pore walls. The cells adhered to each other and were closely connected as they grew along the nanofibers. Cell number was increased compared to the 7^th^ day with the greatest increased for S4. The fiber morphology and scaffold structure changed, and the surface became more fiber-free. S4 and S5 were degraded, and the fiber had agglomerated and had no pore structure. This result was due to the fact that gelatin was soluble in water and degrades faster. The properties of PCL include non-toxicity, biocompatibility and high elasticity.^35^ The PCL membrane elongated under tensile load without premature breaking, so it can maintain the stability of the stent structure during degradation.^36^

Fig. S7† shows a comparison of osteoblast adhesion on S0–S5. Using calcein staining, we showed that the cells adhered to the S1–S5 fibers, and the number of cells on the S4 was higher while the fiber morphology was present. The number of cells increased on day 7 compared to day 3.

### 2.4. Highly active nanofiber composite scaffold promotes osteogenic differentiation

**Fig. 4:**
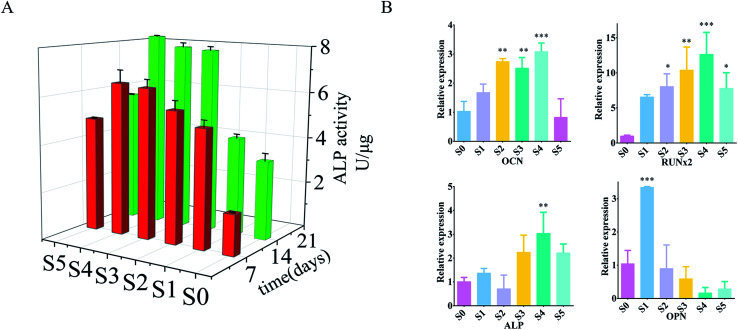
(A) ALP activity after 7 and 14 days of cell and nanofibers culture (B) cell and nanofibers culture for 7 days, PCR measurement of OPN, OCN, ALP, RUNx2 activity. All data were labeled as mean ± standard deviation, *n* = 6, **P < 0.01, ***P < 0.001 compared to S0.

Alkaline phosphatase (ALP) can be used as an early indicator of osteoblast differentiation and mineralization.^37^*In vitro* osteogenic differentiation of osteoblasts on three-dimensional fibers was analyzed by ALP activity assay. The ALP activity of the cells on the three-dimensional fibers after 7 and 14 days is shown in Fig. 4A. At 7 days, within a certain range, the ALP activity of cells carrying sPL on the three-dimensional fiber scaffold was significantly increased; and the ALP activity increased with the increase of sPL. The three-dimensional fiber scaffold carrying sPL was compared with S0 at 14 days. The ALP activity of the cells was significantly increased from the S0 to S5, with the extended time, the ALP activity was significantly enhanced on the 14^th^ days compared to the 7^th^ day.

To further reveal the effect of the three-dimensional nanofiber stent on osteogenic differentiation at the molecular level, we examined the expression of genes involved in a bone formation using RT-PCR on day 14. We checked the expression of calcitonin (OCN), osteopontin (OPN), alkaline phosphatase (ALP), and runt-associated transcription factor 2 (RUNx2) (Fig. 4B) which are responsible for the synthesis of ECM, and Axin2, a regulator of Wnt signaling pathways that plays a vital role in osteogenic differentiation. ALP catalyzed the hydrolysis of phosphates and played a key role in osteoblast activity and bone mineralization.^38^ Quantitative detection of ALP can reflect the differentiation level of osteoblasts with higher activity being indicative of greater differentiation of pre-osteoblasts into mature osteoblasts. OPN was a late marker of osteoblast differentiation.^38^ OCN is a unique marker of osteogenic differentiation that plays a key role in regulating bone formation and mineralization; it is expressed by osteoblast proliferation.^39^ RUNx2 was an early marker of osteoblast differentiation.^39^ RUNx2 was a key transcription factor involved in osteoblast differentiation, which promoted the expression of osteogenic related genes, regulates cell cycle progression, improved bone microenvironment, and affected chondrocytes and the function of osteoclasts.^40,41^ The upregulation of these markers is associated with enhanced osteoblastic activity.^42^ Cells on S1–S4 expressed higher levels of all genes compared to cells on S0. In particular, the ALP and OCN levels of cells on S4 were more than three times higher than S0 (*P* < 0.01), and RUNx2 was increased by 14 fold (*P* < 0.01). S5 also increased, but there was no statistically significant difference between S1, S2, S3, and S5. These *in vitro* evaluations showed that the three-dimensional fiber carrying sPL significantly promoted osteoblast adhesion and proliferation, especially for S4 as it showed obvious promotion of osteoblast proliferation and adhesion.

### 2.5. *In vivo* evaluation of the nanofiber scaffold osteogenic effect

#### 2.5.1. Micro-CT assessment of nano-scaffolds promotes new bone formation and improves osseointegration

**Fig. 5:**
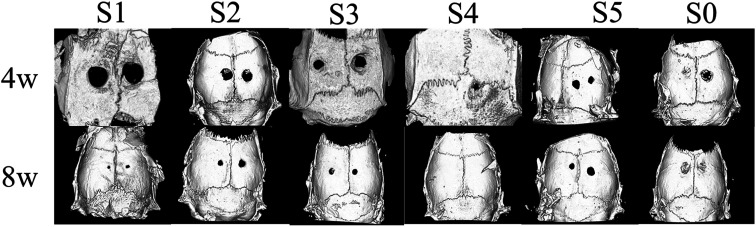
The skull defects of the material treatment group and the control group were scanned at 4 and 8 weeks after surgery. New bone tissue was filled with skull defects covered by sPL-NFS. No defect was observed in other material groups at 4 weeks after surgery. A bone defect was observed in the S4 material group and the other one became smaller. At 8 weeks postoperatively, all bone defects were repaired, and the entire skull defect was filled in the S4 bone tissue, to find a site of significant bone defects; the callus was reconstructed and flush with the bone surface of the untreated site.

To study the application value of nanofibers as fillers for bone defects, we implanted stents in rat skull defects and sacrificed rats at 4 and 8 weeks post-implantation without evidence of any surgical site infection. The bone formation of the samples was evaluated using micro-CT as a non-destructive method. As shown in Fig. 5, nanofibers S2–S5 contains bioactive factors compared to the blank control (S0) or S1 nanofibers. After 4 weeks, the defects of S4 were significantly reduced and one of the bone defects had healed, but the other nanofiber defects had not changed. By the 8^th^ week, all-composite nanofiber-filled bone defects were reduced and had a healing tendency; S4 was completely healed. These results were similar to those achieved by Qi Jiang *et al.*, but unlike their PRP and stem cell combination scaffold, we did not add stem cells in order to avoid the risk of uncertainty in stem cell differentiation.^43,44^ With these results, we speculated that the outer structure was degraded by biological structure and did not induce tissue growth. In the 8^th^ week, the outer layer structure of some fibers was completely degraded, and the material carrying the superactive cytokine within the core had begun to degrade to release growth factors. Bioactive factors induce bone tissue growth and healing. When the defect repairs of S3, S4, and S5 were simultaneously compared, there was no statistically significant relationship between the repair of the bone defect and the material diameter. More importantly, there was no concentration dependence between the results of S1–S5. To further explain these results, it should be considered that sPL was prepared by blood, which contained various growth factor components. Activated platelet lysate released a range of growth factors, such as vascular endothelial growth factor (VEGF) and insulin-like growth factor (IGF), which was higher in concentration than normal blood;^45^ this was essential for bone regeneration. In the above experiments, we have demonstrated sustained release of VEGF and IGF from the scaffold. VEGF can promote chemotaxis^46^ and osteoblast differentiation.^47^ A property that increases bone formation when osteoblasts were presented for 4 weeks.^48^ Exogenous IGF-1 promotes the longitudinal growth of the femur. Besides, IGF can promote bone formation and resorption by directly affecting osteoblasts.^49,50^ Schorn L. *et al.*^51^ found that combining rhBMP-2 and VEGF improved vertical bone formation *in vivo* compared to rhBMP-2 use alone. Therefore, the osteogenesis of sPL can be attributed to the synergy of various growth factors. As for S2, S3, and S5, the defect repair for each group was relatively similar, but S4 has been completely repaired. Our results were similar to previous studies, such as bone tissue engineering materials with PRP for bone defect repair; however, with our modification and the addition of superactive platelet lysate in nanofibers, our scaffold can continuously release various biological factors. Our results also proved that in addition to growth factors within nanofibers, there may be some neurological influence factors that promote the “all or nothing” phenomenon of bone defect repair.

#### 2.5.2. Histological analysis of nanofiber composite scaffolds to promote bone formation

**Fig. 6:**
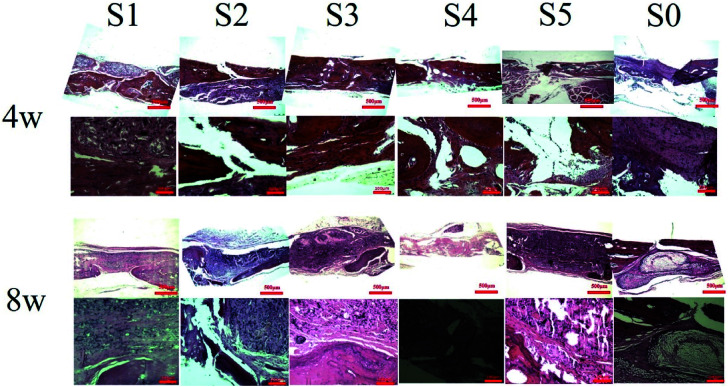
The specimens were treated at 4 and 8 weeks postoperatively for HE staining. In the S4 group, the osteophytes were formed in the defect area, and a small amount of granulation tissue was found in the defect areas of the other material treatment groups and the control group.

**Fig. 7:**
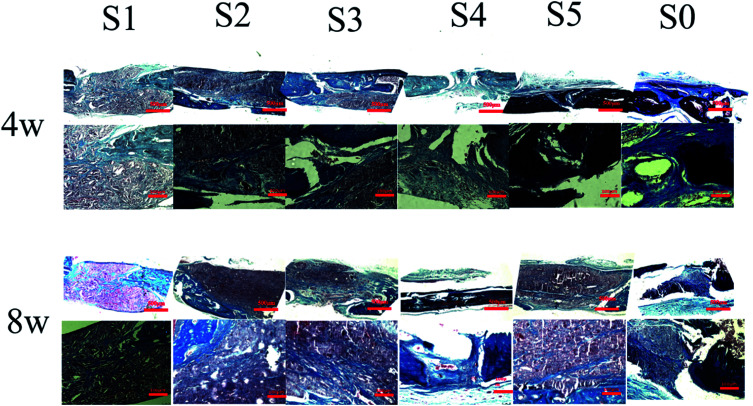
The specimens were treated at 4 and 8 weeks postoperatively for Masson staining. In the S4 group, the osteophytes were formed in the defect area, and only the fibrous tissue was found in the defect areas of the other material treatment groups and the control group.

The *in vivo* biocompatibility of the nanofiber scaffold and bone regeneration within the defect were observed by histological analysis of HE staining and Masson's trichrome staining to obtain more details related to bone regeneration. Each group of histological stains is shown in Fig. 6. At week 4, S1–S3 was implanted, and the defect of S5 was mainly occupied by the stent without any repair by connective tissue. There was no typical bone structure observed for S5, however, new bone formed in the defect with the S4 stent. Nanofibers resulted in larger fibrous tissue block growth due to cellular infiltration from the body, leading to an increased cell density on the randomly oriented fiber structure of PLLA nanofibers. The extent of host cell infiltration and inflammatory cell aggregation around the bone defect covered by the electrospun nanofiber scaffold showed inflammatory cells and a large number of fibroblasts in and around the scaffold. Masson trichrome staining (Fig. 7) further demonstrated a large amount of collagen fibrous tissue and cytoplasm around the bone defect. PLLA did not produce a significant inflammatory response.^52^

After 8 weeks of repair, the regenerated tissue at the defect and the bone tissue surrounding the defect was harvested. HE staining and Masson's trichrome staining (Fig. 6 and 7) showed that there was a small amount of tissue growth around the bone defect of S0–S3, but S5 was larger and the defect was filled. Part of the repair was cellulose, and almost no osteoid tissue was observed. The defects of the S4 treatment group were smaller with the new tissue being collagen fiber and a small amount of cellulose and cytoplasm. It was worth noting that the defects corresponding to the S4 group were almost bridged by the engineered bone tissue bound by the mineralized collagen fibers, revealing that the sPL incorporated three-dimensional nanofibers helped improve the newly formed collagen fiber mineralization. At the same time, nanofibers in sPL can effectively induce new bone formation and tissue mineralization. This result was due to the presence of sPL in these materials. When sPL reached a certain concentration, it promoted the repair of bone defects. The experiment was consistent with the micro-CT analysis. Therefore, we believed that the reprinting of the three-dimensional nanofibers with sPL had an excellent effect of inducing bone growth. In addition, sPL is optimized for the traditional PRP preparation process and has a higher concentration of biologically active factors that can help tissue repair. In the future, we should pay attention to the repair effect of sPL on rat bone defects, but we should not ignore the immunogenicity between different species. Future research will focus on the effects of activated platelets from different species on injury healing.

## 3. Experimental section

### 3.1. sPL production

sPL is performed by centrifuging fresh blood at 1500 rpm for 15 minutes for the first time, discarding most of the bottom red blood cells, centrifuging again at 2800 rpm for 8 minutes, taking the supernatant and repeatedly freezing and thawing at −80 °C/37 °C for 3 times, and then centrifuging again at 3500 rpm for 30 minutes. The supernatant was filtered with a 0.22 μm sterile filter membrane.

### 3.2. Characterization of nanofibers

Through scanning electron microscopy (SEM), water absorption performance, contact angle test, porosity measurement, hemolysis test and biological factor release test, the structure, pore size and storage function of the material for biological factors have been deeply studied.

### 3.3. *In vitro* study

The adhesion of cells to the material was proved by CCK-8, SEM and confocal microscope. The osteogenic properties of the material were proved by PCR and ALP activity detection.

### 3.4. *In vivo* experiment

A bone defect was made on the skull of SD rats, and then spinning materials were implanted. All animal research is conducted with the approval of the laboratory animal ethics committee of The First Affiliated Hospital of Harbin Medical University. The bone defect was repaired by micro-CT and histomorphometric analyses.

## 4. Conclusions

In this study, mineralized gelatin/polylactic acid/polycaprolactone nanofiber membranes with different amounts of sPL were successfully prepared by coaxial co-spinning technology. It was found that the incorporation of sPL could reduce the fiber diameter, increasing the porosity, and promoted osteogenesis. The growth factor release curve indicated that various growth factors in sPL can be continuously released, thereby promoting the proliferation of osteoblasts. Then, osteoblast MC3T3-E1 was inoculated onto a nanofiber membrane containing different amounts of sPL to study the effects of sPL on cell proliferation and osteogenic differentiation. *In vitro* studies showed that as the amount of sPL increased to a certain extent, the nanofiber membrane carrying sPL increased the expression level of ALP activity, the mineralization deposition, and osteogenesis-related markers. Finally, *in vivo* results of the rat skull defect model showed that the S4 nanofiber membrane significantly enhanced bone regeneration as demonstrated by micro-CT and histological staining analysis. Interestingly, the S4 nanofiber scaffolds exhibited excellent osteogenic function and bone defect repair in both *in vitro* and *in vivo* experiments. It was expected to be a highly adaptable local implant material that can be applied in bone tissue engineering. However, electrospinning nanofibers carrying sPL had an “all or nothing” phenomenon in repairing bone defects, which took us to the next step of the study. The mechanism of sPL-loaded nanofibers acting on osteogenesis needs to be further explored and understood. The experimental results showed that the beneficial performance of this bio-structural material paves the way for the design of new tissue-engineered bioactive functional materials, showing a good therapeutic prospect.

## Conflicts of interest

There are no conflicts to declare.

## Supplementary Material

RA-010-D0RA06305C-s001
