# Supplementary material for: Coaxial nanofiber scaffold with super-active platelet lysate to accelerate the repair of bone defects
Source: RSC Adv. 2020 Sep 29;10(59):35776–86. doi: 10.1039/d0ra06305c (PMC9056889; doi:10.1039/d0ra06305c)
Supplement: RA-010-D0RA06305C-s001 [file RA-010-D0RA06305C-s001.pdf]

Electronic Supplementary Material (ESI) for RSC Advances.
This journal is © The Royal Society of Chemistry 2020

Coaxial nanofiber scaffold with super-active platelet lysate to accelerate the repair of bone defects

Zhipeng Huang[1], Wantao Wang[1], Qinglong Wang[1], Taylor Hojnacki[2], Yanli Wang[1], Yansheng Fu[3], Prof. Wenbo Wang[1*]

1. The First Affiliated Hospital of Harbin Medical University, 23 You Zheng Street, Harbin 150001, China

2. Department of Cancer Biology, Abramson Family Cancer Research Institute, Perelman School of Medicine, University of Pennsylvania, 421 Curie Blvd., Philadelphia, PA, 19014, USA

3. Tianqing Stem Cell Co., Ltd., Jubao Second Road, Science and Technology Innovation City, Songbei District, Harbin, 150000, China

*Corresponding authorName: wenbo wang e-mail: wenbowang1967@163.com

Support information

1. Materials and Methods

Ester-sealed polylactic acid and Ester-capped polycaprolactone were purchased from Jinan Daigang Biomaterial Co., Ltd, (Shandong, China). 2,2,2-trifluoroethanol (≥99.8%) and gelatin were purchased from Shanghai Aladdin Reagent, China. Other experimental research reagents were purchased from Solarbio®(Beijing, China). All other chemicals were of analytical grade.

1.1 Fabrication of three-dimensional nanofiber scaffolds

The three-dimensional nanofiber scaffolds was prepared by coaxial electrospinning and freeze drying. 0.4 g of gelatin and 0.6 g of PLLA were separately dissolved in 5 ml of 2-2-2 trifluoroethanol, and then a precursor solution was prepared by mixing a gelatin solution with a PLLA solution at a volume ratio of 4:1. The precursor solution was placed in a 10 ml syringe and attached to the outer circumference of the shaft electrospinning tube.

0.5 g of PCL pellet was added to 5 ml of the solution and stirred overnight. Then, the 5mL was equally allocated into 5 tubes. We added 0 μL, 100 μL, 300 μL, 600 μL, and 900 μL of sPL liquid to the tubes and labeled them S1, S2, S3, S4, and S5, respectively. The following parameters were utilized for electrospinning: Core: 0.5mL/h, Shell: 2.1 mL/h; Positive voltage: 12.85±0.45kv, Negative voltage: 1.67±0.2kv, Temperature: 22.5 ± 3°C, and Humidity: 55.4 ± 8.2%. These parameters were optimized for different components for continuous electrospinning and for the production of beadless fibers. The spun fiber were dried under the freeze dryer to form a three-dimensional scaffolds .(Shanghai Lanyi). sPL is based on the traditional ultra-low temperature freeze-thaw preparation technology of platelet-rich lysate, and uses patented cytokine culture technology to cultivate ordinary PL by special in vitro high-efficiency induction, activation, and constant temperature carbon dioxide cell incubator to achieve platelet lysis. There are more biologically active factors with higher

concentration in the liquid and better activity. At the same time, specific biologically active factors such as PDGF, TGF-β, IGF, EGF, VEGF and FGFs are specifically induced.

1.2 scaffolds characterization

1.2.1 Morphological structure of nanofibers

Surface morphology was observed using a scanning electron microscope. The fiber diameter was measured from the scanning electron microscope image by Image-J, and at least 10 fibers were measured per sample.

The morphology of the fibers was observed by SEM after the scaffolds were degraded after 14 days in the PBS at 37 °C. A transmission electron microscope was used to observe the internal structure of the fiber.

1.2.2 Water absorption performance test

The dry three-dimensional scaffold was weighed, and the mass at W0 was recorded. The scaffold was then immersed in deionized water. Timing began after the scaffold were placed in the deionized water. The scaffolds were removed after 5 min, 15 min, 30 min, 60 min and 120 min respectively. We removed the scaffolds from the water and weighed and recorded the mass (Ww) in wet state. Water absorption rate (W) was calculated according to formula (1)[53]: An average of three parallel samples was calculated for each group.

$$W = (W_w - W_0)/W_0 \times 100 \qquad (1)$$

1.2.3 Water contact angle measurement

The hydrophobicity of the scaffold was analyzed using the SSIELDEROP water contact angle measurement surface analysis system. A drop of water was placed on the surface of the nanofiber scaffold and the contact angle was measured after 5 seconds at room temperature.

The water absorption properties of nanofiber scaffolds are beneficial for biomedical applications. As shown in Figure S2, the S1-S5 scaffold is placed in a glass dish containing a sufficient amount of water. S1 exhibits a slower water absorption rate, which is reached maximum water absorption after 60 min; S2-5 stents all exhibit rapid water absorption properties, and the maximum water absorption can be achieved by putting them in water for 15 minutes. Between S1-S3, the maximum water absorption rate increases slightly with the increase of sPL in the scaffold; between S3-S5, with the increase of sPL, the water absorption rate decreases, and the scaffold grows with time. The maximum water absorption rate did not change much, demonstrating that the stent exhibited strong water absorption properties. The reason is that the main constituent materials of the stent are PLA and gelatin, and the gelatin has a large water absorption property, and the hydrophilicity is remarkably increased. In addition, different sized holes in the stent also facilitate water absorption. As the sPL in the stent increases, the water absorption rate decreases to some extent. This may be because the sPL increases, hydrophilic substances such as gelatin absorb water quickly to reach saturation. and therefore the water absorption capacity decreases. This is somewhat different from the measurement of the contact angle, probably because the contact angle measures the result of water being contacted 5" on the spin film, and hydrophilicity is the result of measuring the complete water absorption of the spun material.

1.2.4 Composite scaffold material porosity determination

We utilized the porosity detection method according to Serra IR et al.[54]: Weigh the mass (W0) of the stent in the dry state, and immerse the stent in absolute ethanol for 2 h. Then weigh the mass (W1) of the stent again, according to formula (2) Calculate the porosity of the stent ρ:

$$\rho=(W_1-W_0)/(\rho\, V_{steady})\times 100 \qquad (2)$$

ρ is the density of absolute ethanol

1.2.5 Hemolysis analysis

According to previous studies[55], New Zealand white rabbit blood with anticoagulant was centrifuged at 500g for 10 minutes at 4℃. The RBC pellet was washed with physiological saline until the supernatant no longer contained any hemoglobin observed by the naked eye. The RBC suspension was then diluted 1:5 in PBS and 1 ml of the dilution was mixed with 1 mg of polymer. The saline group was used as a negative control (0% hemolysis), and deionized water was added as a positive control (100% hemolysis). After incubating at 37℃ for 1 hour, the plate was centrifuged at 500g for 10 minutes at 4 ℃. 100 μL was taken and added to a 96-well plate, and the OD value was measured at 520 nm using a microplate reader. The hemolysis rate was calculated using the formula (3). Measurements were given as the average of three parallel samples for each group.

Hemolysis rate = (test - negative) / (positive - negative) ×100% （3）

1.2.6 Growth factor release was studied by detection of IGA and VEGA

10mg of the material was weighed and added to 500μL of deionized water. The liquid was removed at 1, 4, 7, 15, 30, and 40 days and stored at -80 °C for use. According to the manufacturer's instructions, the amount of IGA and VEGA released was evaluated by an ELISA kit.

2 In vitro study of the interaction between three-dimensional nanofiber materials and cells

2.1 Cell viability evaluation

S1, S2, S3, S4, and S5 were made into discs having a diameter of about 6 mm. Each disc was then disinfected with Ethylene oxide and washed twice with PBS. We then soaked the scaffold with DMEM-high glucose complete medium (containing 10% fetal bovine serum and 1% double anti-penicillin/streptomycin) for 24h in a 96-well plate. A cell suspension of 1×10^5 MC3T3-E1 was added to each well, and three parallel samples were made for each group. CCK-8 cells were added at 4h, 8h, 24h, 72h, and

168h after plating. Cell adhesion was measured by OD value and cell proliferation rate was calculated using equation (4). Each experiment was repeated three times.

$$\text{Cell viability (\%)} = [A]_{test} / [A]_{control} \times 100\% \quad (4)$$

2.2 Cell adhesion and proliferation observation

The cells were placed on the scaffold material, and the medium in the well plate was aspirated on the 3rd and 7th day after implantation. The cells were washed with PBS 3 times and stained with calcein to observe cell adhesion by confocal microscopy.

For cell growth, cells were cultured for 3, 7, and 14 days. To asses proliferation, the scaffolds were washed 3 times with PBS, then immersed in 10% glutaraldehyde fixative for 60 min. The fixative was washed off followed by a PBS rinse 3 times. The scaffold was then dehydrated with gradient alcohol (30%, 50%, 60%, 70%, 80%, 90%, 100%), and each concentration of alcohol was dehydrated for 10 min in the freeze dryer. After the lyophilized stent was sprayed with gold, the cell growth on the stent was observed under SEM.

In the same manner, cells were fixed with a 4% paraformaldehyde solution (Sigma, US) for 10 minutes at room temperature. Cells were permeabilized with 0.5% (v/v) Triton X-100 for 5 minutes and washed again with PBS. We labeled cell viability with 0.5 mL of 5 μg/mL FITC-phalloidin (solarbio, China) and 0.5 mL of 10 μg/mL 4,6-diamidino-2-phenylindole dialectic acid (DAPI, solarbio) for protein and nucleus detection, respectively. Cell adhesion was observed under a confocal microscope (Leica).

2.3 Alkaline phosphatase measurement

The cell and material culture methods were the same as above. Blank control wells, standard wells, and sample wells were set using a 96-well plate. Cells were lysed with 1 ml of cell lysate (Solebao) at 7 and 14 days, respectively, and the samples were frozen at -80 ° C. The 50 μl sample was mixed with of detection buffer or chromogenic

substrate and incubated at 37 ° C for 10 minutes. 100 μl of the reaction stop solution was added to each well to terminate the reaction. We measured the absorbance at 405 nm. The alkaline phosphatase activity in the sample was calculated based on the enzyme activity definition. The experiment was repeated three times.

2.4 Evaluation of osteogenicity by the polymerase chain reaction

After the cells were co-cultured with the coaxial spinning fibers for 14 days, all the culture solutions were aspirated, and the cells were lysed with TrIzol^{TM} Reagent (Invitrogen). Total RNA was extracted according to the reagent instructions. Briefly, chloroform was added to each sample, and the sample was centrifuged to separate the phases. The precipitated RNA particles were washed in 75% ethanol, then resuspended in RNase-free water. The PrimeScriptTMRT reagent kit with gDNA Eraser (Takara) was mixed to prepare cDNA. The real-time PCR analysis of gene expression was performed using an ABI Prism 7000 Sequence Detection System (Applied Bio.). The glyceraldehyde-6-phosphate dehydrogenase (GAPDH) primer/probe set, Run2, ALP, OPN, and OCN primers were derived from Li Mei et al [56](Table 1). The reaction was carried out in a 96-well plate with a final capacity of 20 ml. For each sample, a cycle threshold (Ct) was obtained and the replicated values were averaged. The relative expression of each target gene was then calculated using the $2^{-\Delta\Delta CT}$ method. The experiment was repeated three times.

Table 1 Primers sequence used for evaluation of the osteogenic effect

**Gene**	**Primers sequence**	
	**Forward**	**Reverse**
ALP	5′-GCTACACCACAACACGGGCGA-3′	5′-TCCAAATGCTGATGAGGTCCA-3′
OPN	5′-TGAGACTTGCAGTGATTTGCTTTTGC-3′	5′-CTCATCTGTGGCATGGGGATACTG-3′
GAPDH	5′-ACCACAGTCCATGCCATCAC-3′	5′-CCACCACCCTGTTGCTGTA-3′
RUNX2	5′-TCCAACCCACGAATGCACTA-3′	5′ -GAAGGGTCCACTCTGGCTTTG-3′

OCN	5′-CAATAAGGTAGTGAACAGAC-3′	5′-CTTCAAGCCATACTGGTCT-3′

2.5 In vivo evaluation of osteogenic effects of three-dimensional nanofiber materials

All animals were provided by the Animal Experimental Center, the Second Affiliated Hospital of Harbin Medical University. This study was approved by the Ethics Committee of Laboratory Animal Ethics Committee of the First Affiliated Hospital of Harbin Medical University (No. 2019029).

The 10-week-old male Sprague-Dawley rats weighing 200-250 g were randomly divided into 6 groups. After a 50mg/kg intraperitoneal injection of 3% pentobarbital sodium, the rats were anesthetized with 1% lidocaine, and the skin was cut along the midline of the skull. The skin, soft tissue, and periosteum were separated layer by layer, and the parietal bone was exposed. In the flattest part, a 3.0 mm full-thickness circular skull defect was made on both sides of the cranial. We were very careful not to damage the dura mater. Self-curing calcium phosphate（S0）and five different experimental materials were implanted, and the periosteum, soft tissue, and skin were sutured layer by layer. After the rats were awake, they were placed in a cage. The animals were observed daily for activity, diet, mental state, and incision healing. The rats were sacrificed 4 and 8 weeks after surgery, and the tissues were fixed with 4% paraformaldehyde. Micro-CT scans (SkySCA1176, Belgium) were used to evaluate the regeneration of new bone in the defect area. All skull specimens were demagnetized with 10% EDTA after micro-CT scan, dehydrated with a graded ethanol solution, embedded in paraffin, cut into 5 μm sections, and histologically observed by HE and Masson trichrome staining. All staining photos were taken with a fluorescence microscope (Leica).

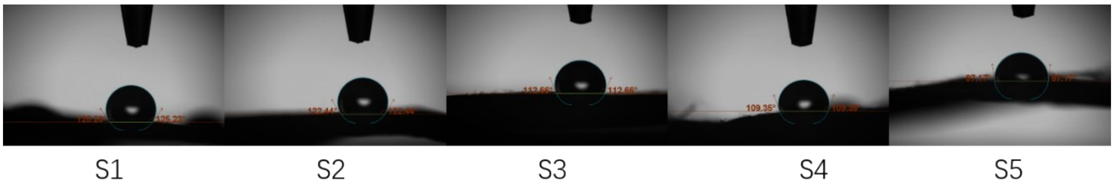

Figure S1 Water Contact Angle Diagram

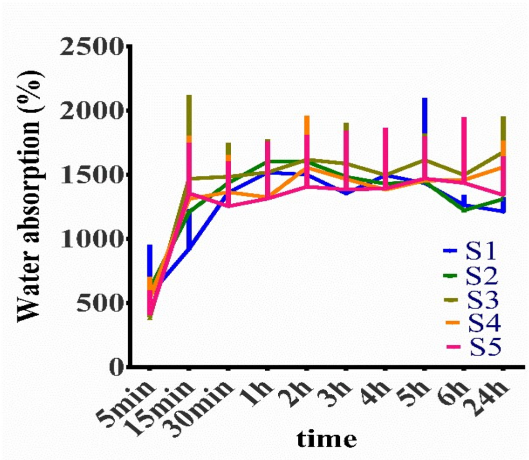

Figure S2 Water absorption of sPL composite scaffold material loaded

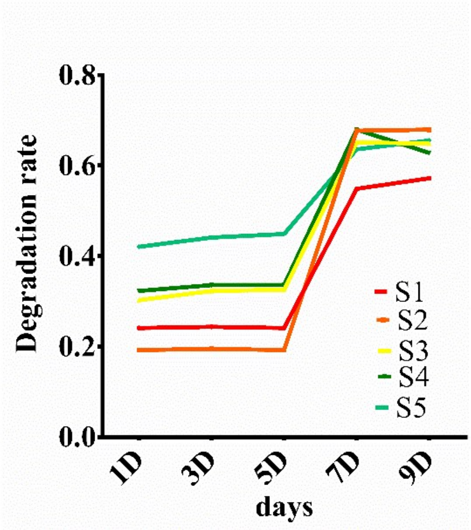

Figure S3a Degradation of nanofibre composite scaffolds

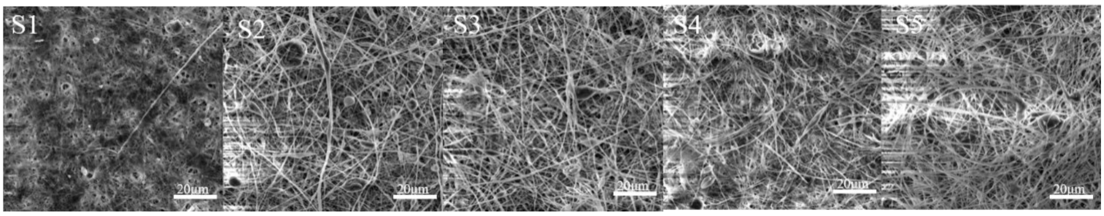

Figure S3b Degradation of nanofibre composite scaffolds in 14 days by SEM

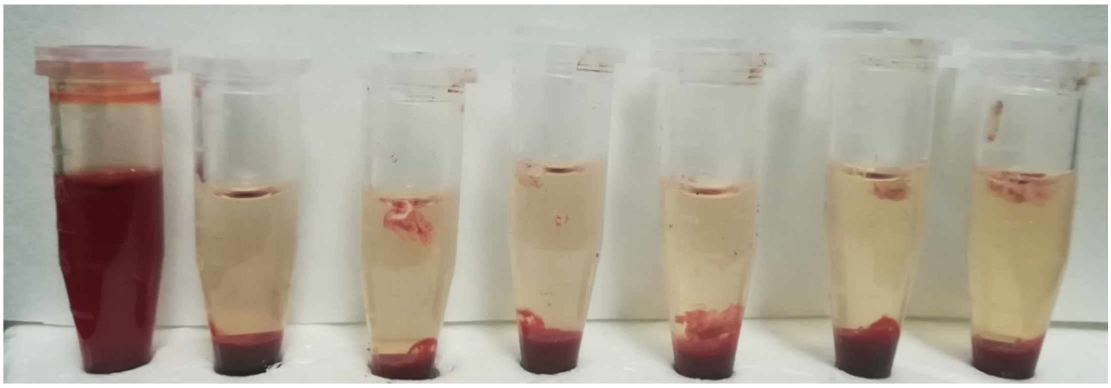

Figure S4 Nanofiber composite scaffold hemolysis experiment picture

7D

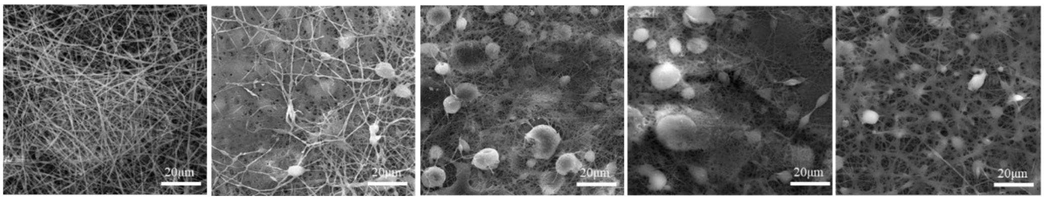

Figure S5 Results after 7 days of cell co-culture with sPL three-dimensional fiber scaffold

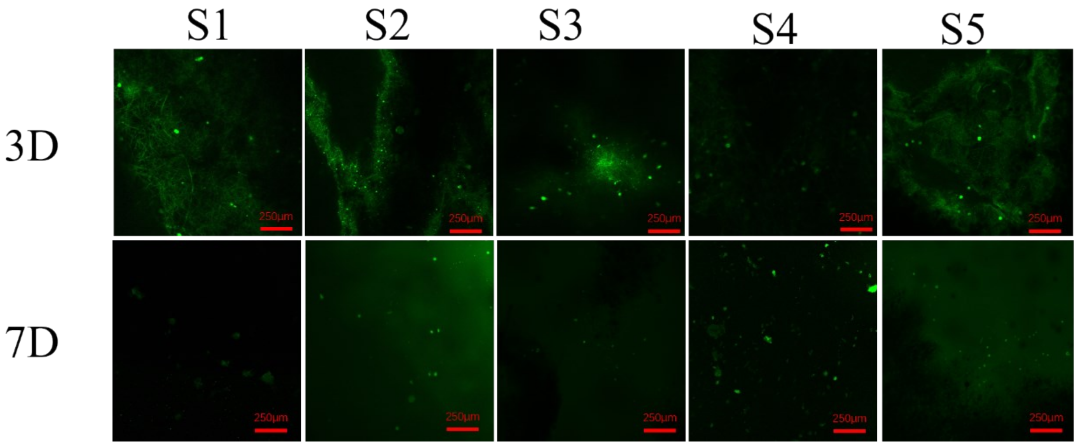

Figure S6 Staining results of calcein on nanofiber composite scaffolds and cells after 3 and 7 days

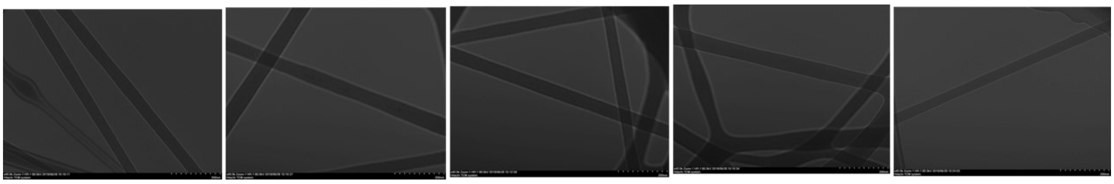

Figure S7 The nanofiber scaffolds are S1, S2, S3, S4, S5 from left to right
